# Management of mobile thrombus of the thoracic aorta

**DOI:** 10.1016/j.jvscit.2021.07.009

**Published:** 2021-08-27

**Authors:** Yash P. Vaidya, Tonio F. Schaffert, Palma M. Shaw, Michael J. Costanza

**Affiliations:** a Department of Surgery, SUNY Upstate Medical University, Syracuse, NY; b Department of Vascular Surgery, SUNY Upstate Medical University, Syracuse, NY

**Keywords:** Thoracic aortic mobile thrombus, Thoracic endovascular aortic repair, Floating thrombus

## Abstract

Mobile thrombus of the nonaneurysmal, nonatherosclerotic aorta is a rare condition but presents with catastrophic embolic events. We describe two cases that demonstrate differences in presentation and treatment strategies. We review the literature to discuss initial management as well as surgical options. However, due to the limited number of cases, no definitive guidelines for management exist.

Symptomatic thoracic aortic floating thrombus is an unusual but significant source of cerebral or peripheral arterial emboli. The usual workup for symptomatic emboli involves imaging modalities to evaluate for a cardioembolic source. The availability of transesophageal echocardiogram (TEE) or computed tomography angiography (CTA) has resulted in more frequent diagnosis of thoracic aortic sources. Intravenous therapeutic anticoagulation is the initial treatment with a transition to oral anticoagulation or intervention depending on the clinical situation. Because past experience with thoracic floating thrombus is limited to case reports and small case series, no clear guidelines for management exist. We describe our experience with two cases that demonstrate different management approaches. Informed consent from the respective patients to publish their case details and images has been obtained.

## Description of cases

### Case 1

**Fig 1:**
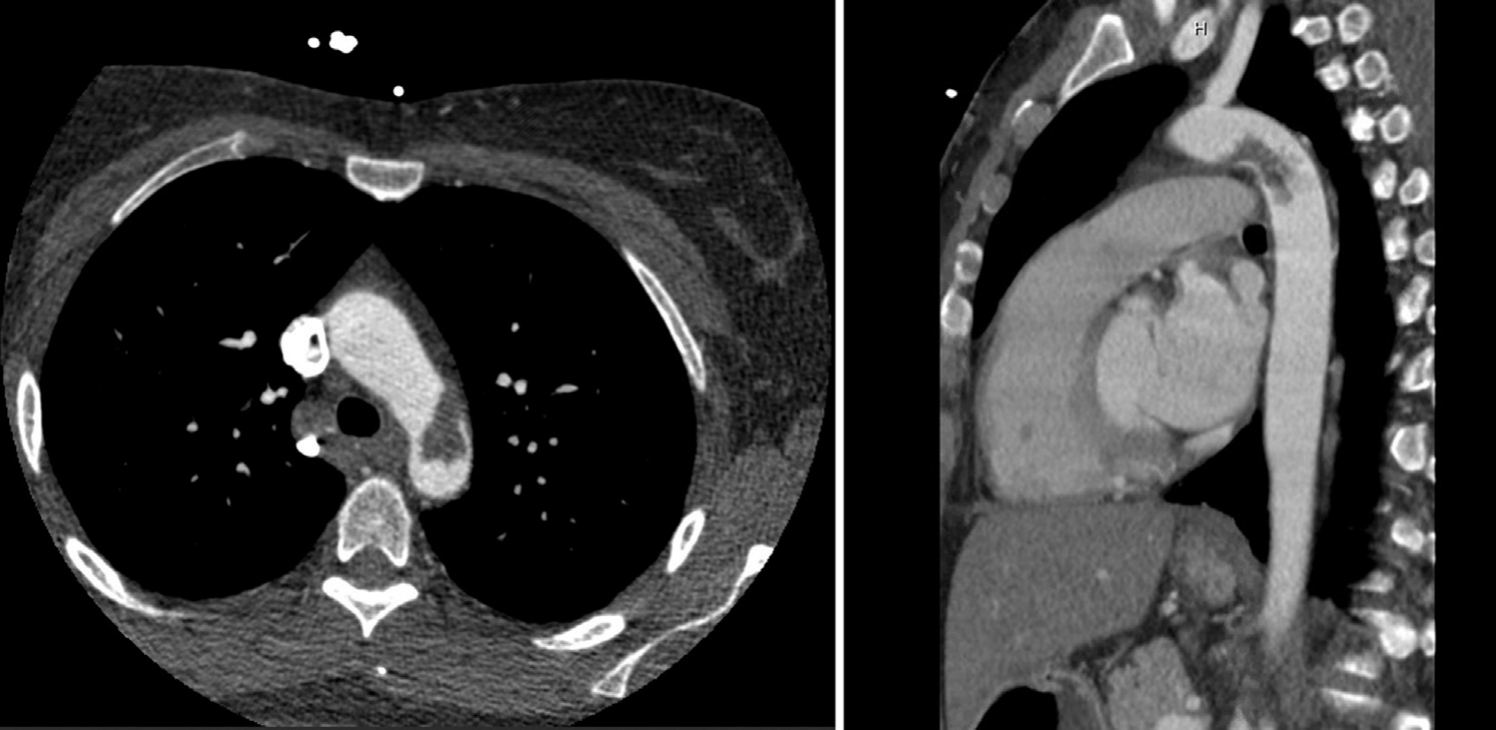
Axial and sagittal views showing the mobile thoracic aortic thrombus immediately distal to the left subclavian artery origin.

A 47-year-old woman presented with acute onset of neurological symptoms that included a vague “pop” in her head along with left upper extremity numbness and left monocular vision loss. A CTA head and neck showed patent intra- and extracranial vasculature without evidence of dissection. A CTA thorax showed a mobile thoracic aortic thrombus immediately distal to the left subclavian artery origin (Fig 1). She also had evidence of small splenic infarcts on CTA abdomen/pelvis. The splenic infarcts were stable from her presentation for abdominal pain the previous year, when workup had eliminated the possibility of cardiac vegetations.

Her magnetic resonance imaging brain showed multiple, bilateral punctate cerebral, and cerebellar infarcts. Transthoracic echocardiogram was normal without left atrial thrombus. Coagulation studies including prothrombin time/international normalized ratio, partial thromboplastin time, protein C and S activity, lupus anticoagulant, prothrombin mutation, anticardiolipin antibodies, and beta 2 glycoprotein were within normal limits.

**Fig 2:**
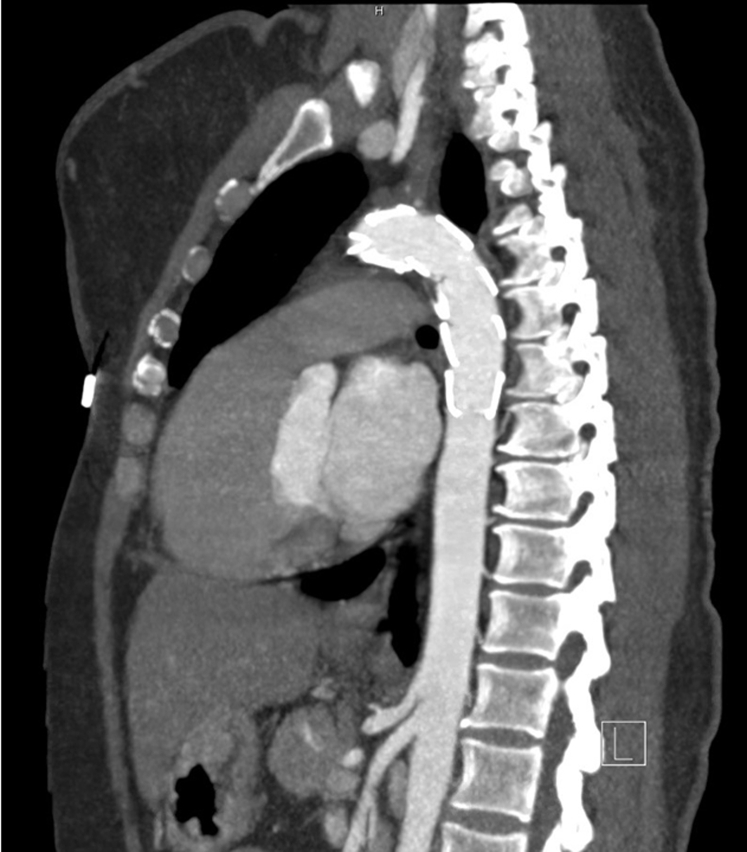
Adequate position of the stent graft with resolution of the thrombus at 18 months.

After therapeutic anticoagulation with heparin, she was treated with thoracic aortic stent grafting to exclude the aortic thrombus and a left carotid-subclavian transposition to obtain an adequate landing zone for the graft. The decision to proceed with this intervention was based on the fact the she had neurologic symptoms with imaging-confirmed infarcts and no embolic source other than her mobile, thoracic aortic thrombus. There were no perioperative complications, and her 18-month follow-up imaging shows a satisfactory position of the thoracic stent graft with a patent carotid subclavian transposition (Fig 2).

### Case 2

A 53-year-old woman with a history of cocaine abuse presented with global aphasia and right hemiparesis. She was diagnosed with a left middle cerebral artery territory ischemic stroke and NIH Stroke Scale/Score 22. She had an emergency transfemoral cerebral angiogram with mechanical thrombectomy of her left internal carotid artery embolus, which achieved thrombolysis in cerebral infarction recanalization. After the embolectomy, she developed worsening cerebral edema, with early evidence of uncal herniation on CT head requiring a decompressive craniotomy.

**Fig 3:**
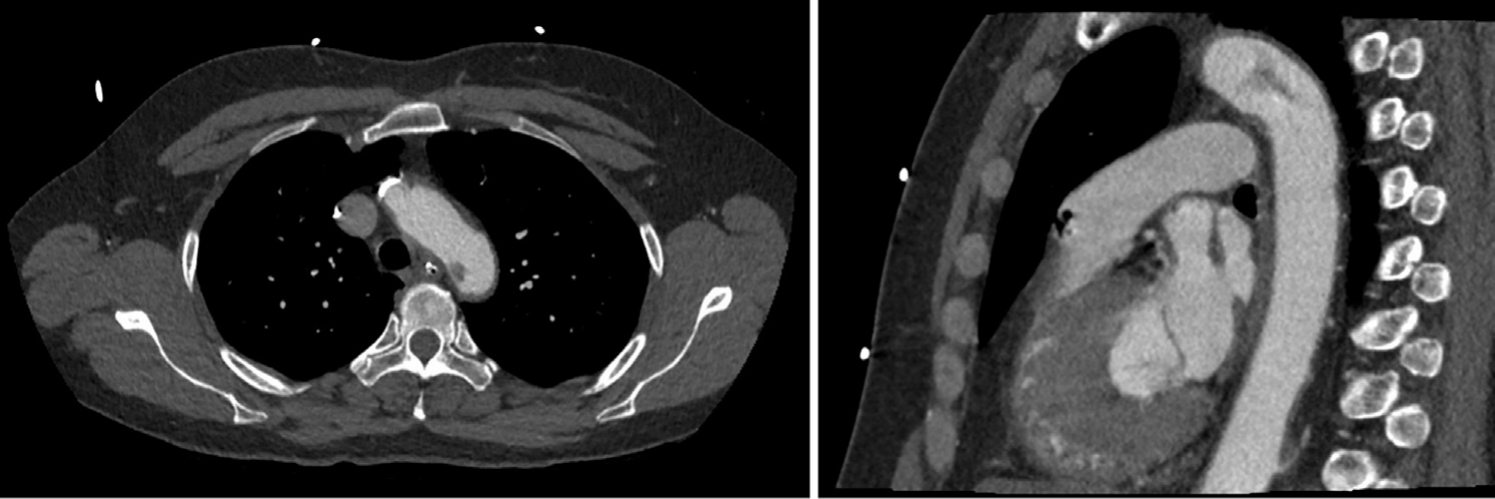
Axial and sagittal views demonstrating a mobile thrombus at the medial wall of the aortic arch.

During her evaluation, a CT angiogram of the neck and thorax showed a mobile thrombus of the medial wall of the aortic arch, distal to the origin of the left subclavian artery (Fig 3). TEE confirmed a 2 cm mobile atheroma of the distal aortic arch, without left atrial thrombus, confirming the source of the embolic event. There was no evidence of hypercoagulability on workup.

Because of her guarded neurological prognosis, noninterventional therapy was initiated in the form of therapeutic anticoagulation with unfractionated, intravenous heparin. Repeat imaging studies 25 days after her initial presentation showed complete resolution of the aortic thrombus.

## Discussion

Floating thoracic aortic thrombus is a rare occurrence, although the liberal use of imaging studies has increased the detection of this condition. In their study on 10,671 autopsies, Machleder et al^1^ noted a 0.45% incidence of aortic mural thrombus. Of these cases, 17% had distal embolization on autopsy, with 6% noted to have major thromboembolic occlusions related to the cause of death.^1^

Aortic thrombus noted in a nonaneurysmal, nonatherosclerotic descending thoracic aorta (NAADTA) has been described as a distinct clinical entity. Although conditions predisposing to atherosclerosis contribute to the pathogenesis, a high prevalence of hematologic diseases and hypercoagulable conditions has been reported.^2^

Despite multiple descriptions of aortic thrombus, diagnostic criteria do not exist for this condition. TEE can help surgical planning by providing high-resolution images of the aortic wall and base of the mural thrombus. Although TEE can underestimate the size of the thrombus, the dynamic nature of the modality can detect thrombus mobility. Although being less sensitive than TEE, CTA has the ability to image the abdominal aorta and define the embolic source.^3^ Yang et al^4^ described a parameter termed break-off risk ratio, a ratio of the length of the floating and attached portion of the lesion, as an indicator of its potential to embolize.

Because of scarcity of available data and experience, no specific treatment guidelines exist for the management of mobile aortic thrombus. In several reports, therapeutic anticoagulation successfully resolved the thrombus.^5^^,^^6^ The success of anticoagulation has been attributed to the high prevalence of coagulation abnormalities in patients with floating aortic thrombi and NAADTA. Despite these reports, there are no widely established recommendations on the use of either anticoagulants or antiplatelet agents.

Choukroun et al^7^ recommended reserving surgery for patients who have persistent thrombus on TEE after 2 weeks of therapeutic anticoagulation with heparin. In contrast, a recent meta-analysis recommended surgery as primary treatment of floating aortic thrombus in NAADTA. The authors reported that anticoagulation seemed to be associated with higher rates of recurrence, complications, and limb loss if used as primary therapy.^8^ Surgical management in most of the earlier reports consisted of open aortic thrombectomy using cardiopulmonary bypass and hypothermic circulatory arrest. These reports recommended early surgical intervention due to low operative risk of complications and its role in preventing fatal recurrent embolic events.^9^^,^^10^

There are several recent reports that describe favorable outcomes with thoracic stent grafts.^11^, ^12^, ^13^ Despite the theoretical risk of embolization from wire manipulation and graft deployment, no complications have been reported. It is important to ensure complete entrapment of the thrombus between the stent graft and aortic wall. Tsilimparis et al^14^ recommend oversizing the stent graft by 5%-10% to obtain coverage of at least 2 cm both proximal and distal to the longitudinal extension of thrombus. They also recommend endovascular repair for mobile pedunculated thrombus, or sessile thrombus with recurrent embolic events. Open surgery is reserved for patients not amenable to the endovascular approach and those with suspected malignancy.^14^

## Conclusions

Thoracic aortic floating thrombus is rare but dangerous source of systemic emboli resulting in significant morbidity. No clear guidelines exist for management. Therapeutic systemic anticoagulation is the initial treatment; however, early surgical intervention has excellent reported results. Both endovascular and open surgical techniques have been deemed safe, and definitive treatment should be individualized.
